# Trends in unsuitability for blood donation in the Brazilian Amazon

**DOI:** 10.3389/fpubh.2022.1056332

**Published:** 2022-12-22

**Authors:** Graciela Marleny Rivera Chavez, Anderson Nogueira Barbosa, Gemilson Soares Pontes

**Affiliations:** ^1^Programa de Pós-Graduação em Hematologia, Fundação Hospitalar de Hematologia e Hemoterapia do Amazonas, Universidade do Estado do Amazonas (UEA), Manaus, Amazonas, Brazil; ^2^Laboratório de Virologia e Imunologia, Coordenação de Sociedade, Ambiente e Saúde, Instituto Nacional de Pesquisas da Amazônia (INPA), Manaus, Amazonas, Brazil

**Keywords:** blood donation, blood donor, clinical screening, donor unsuitability, Brazilian Amazon

## Abstract

**Introduction:**

Sociodemographic and behavioral factors are usually associated with the unsuitability to donate blood. Understanding the reasons behind the exclusion of blood bags is crucial for reducing donor deferral rates. This study aimed to characterize the profile of unsuitable donors in the Blood Center of the northern Brazilian state of Amazonas.

**Methods:**

This is a retrospective study, based on documentary analysis of electronic medical records obtained in the Hematology and Hemotherapy Foundation of the state of Amazonas. This study included all individuals with complete medical records (*n* = 87,463) who tried to donate blood between 2017 and 2019.

**Results:**

The overall rate of donor unsuitability was 19.12% (*n* = 16,627) and the main reason was poor nutritional status (15.17%), followed by chronic health problems (11.40%), risky sexual behavior (9.5%) and exposure to risk (8.83%). High blood pressure figured as the leading cause of unsuitability among chronic health conditions (85.19%), while having sex with multiple partners (92.63%) was the main sexual risk behavior. The risk of exposure to malaria was responsible for 99.45% of unsuitability among those who were unsuitable due to exposure to risk factors. Deferral rates were associated with increasing age and replacement donation, which was the predominant type of donation. Women had the highest rates of unsuitability, mainly during the first donation (37.04%).

**Discussion:**

This study provides the first known profile of blood donor unsuitability in the Brazilian Amazon and raises awareness of the region-specific needs that must be met to reduce blood donor unsuitability.

## 1. Introduction

The evolution of blood donation is marked by a trajectory full of social, political and cultural factors that allowed the construction, transformation and growth of the practice of transfusion (1). Epidemiological situations have influenced transfusion practice over time by eliciting analyses and reflections in moments of social crisis from the socioeconomic, sanitary and cultural reality of society (1, 2). The greatest example is the emergence of the acquired immunodeficiency syndrome (AIDS) pandemic during the 1980s (3).

Caused by the human immunodeficiency virus (HIV), AIDS is considered one of the major public health problems, and contributed to the recognition of the possibility of transmission of pathogens through the blood. From this moment on, drastic changes were made in the management of the entire donation process and transfusion practice (3, 4). In this context, it became necessary to develop regulatory instruments for the practice of blood donation that involve public policies, legislation and actions that ensure the quality of processes, in order to improve transfusion safety (5). However, it is important to emphasize that even with the implementation of several protocols, serological screening does not provide complete safety regarding the possibility of transmission of pathogens *via* blood transfusion (5, 6).

One measure to maintain transfusion safety is the selection and exclusion of donors through clinical screening. This procedure aims to investigate epidemiological and behavioral aspects that no laboratory test is able to identify, which improves the safety in the search for volunteers and, consequently, the quality and safety of donated blood (7, 8). Nonetheless, the rigidity of clinical screening based on current Brazilian rules and legislation can also be a limiting factor for blood donation (9). High rates of temporary postponement of candidates for donation or exclusion of donors, an increase in the rate of unsuitability and probably a deficit in blood bank stocks are all related to the rigidity of clinical screening of donors (5, 7, 9).

In Brazil, studies evaluating the unsuitability and exclusion of blood donors are scarce and not comprehensive. Most studies focus only on exclusion or unsuitability due to positive test results for infectious diseases, such as the study conducted in Ribeirão Preto that found that hepatitis C was the most frequent infection (56%) among unsuitable donors, followed by hepatitis B (20%), HIV (approximately 12%) and syphilis (10%) (10). However, there are still few studies that evaluate the impact of clinical screening on the rates of unsuitable candidates (9, 11). Defining the profile of donors and the factors that are associated with their unsuitability is of great importance in order to direct the implementation of corrective measures and encourage the return of temporarily unsuitable donors for new donation and retention.

The northern region of Brazil, especially the state of Amazonas, has socio-demographic, economic and epidemiological peculiarities that can considerably influence the rates of unsuitability of blood donors. This study aimed to evaluate the profile of unsuitable donors in the state of Amazonas in the period 2017–2019 in order to obtain data that allow us to improve the process of capture, selection and screening of donors.

## 2. Materials and methods

### 2.1. Type of study and ethical aspects

This is a retrospective observational study, based on documentary analysis of the virtual medical records of the donor sector at the Hematology and Hemoterapy Foundation of the state of Amazonas (HEMOAM). The study was duly approved by the Research Ethics Committee (REC) at HEMOAM (CAEE 39449920.3.0000.0009), in accordance with Resolution No. 466 of December 2012 of the Brazilian National Health Council. Considering the precepts stipulated for the use of documentary data, there was no need for an informed consent form (ICF). The information collected was solely for scientific purposes and secrecy and confidentiality procedures were ensured during the use of the data.

### 2.2. Study population

This study targeted information regarding all donors who presented with the intention of donating blood at HEMOAM in the period between 2017 and 2019. The sampling was non-probabilistic and consecutive.

To compose the sample, the information from suitable and unsuitable donation candidates was used. This information is contained in the electronic records of the donors who attended the HEMOAM blood bank from January 2017 to December 2019, and any donors who had gone for more than one year without donating were considered inactive. The volunteers were identified only by their registration number in the HEMOSYS (Hemocenter Management System) software to preserve the confidentiality of their personal data. Individuals with incomplete medical records or inconsistent data were excluded from the sample. A total of 87,463 individuals were included in the final sample of this study. Data were collected between December 2020 and March 2021.

### 2.3. Data collection

For the collection, sociodemographic data, quantity of donations, type of donation, donor ID, sex, age, level of education and marital status were used. All the obtained data had been registered in the HEMOSYS program. Regarding the aspects of hematological and clinical screening, data points, such as weight and hematocrit, were analyzed, as well as the standardized clinical data questionnaire that is applied and which contains questions regarding infectious diseases, chronic diseases, acquired diseases, and risk habits, etc. All data collected refer to donations made in the period stipulated in the study.

### 2.4. Statistical analysis

The data collected were initially tabulated and filtered using the Microsoft Excel 2019 program. During filtering, only donors whose data was completely filled in were considered. Sociodemographic variables and reasons for clinical unsuitability, as well as values of hematological unsuitability, self-exclusion and confidential exclusion were analyzed in a descriptive way through absolute and relative frequencies, taking into account first-time donations and return visits. In the search for factors associated with clinical unsuitability, sociodemographic variables were analyzed using the odds ratio (OR) analysis calculated by logistic regression using SPSS Statistic program (version 25), with a 95% confidence interval (CI). The tests ANOVA, Student's t and Pearson's correlation were used for normally distributed data. Strong positive correlations were considered as r >0.7 (12). These statistical and graphical tests were performed using the program GraphPad Prism version 8.0.1. The significance level was set at 0.05.

## 3. Results

### 3.1. Unsuitability profile according to sociodemographic characteristics

**Figure 1:**
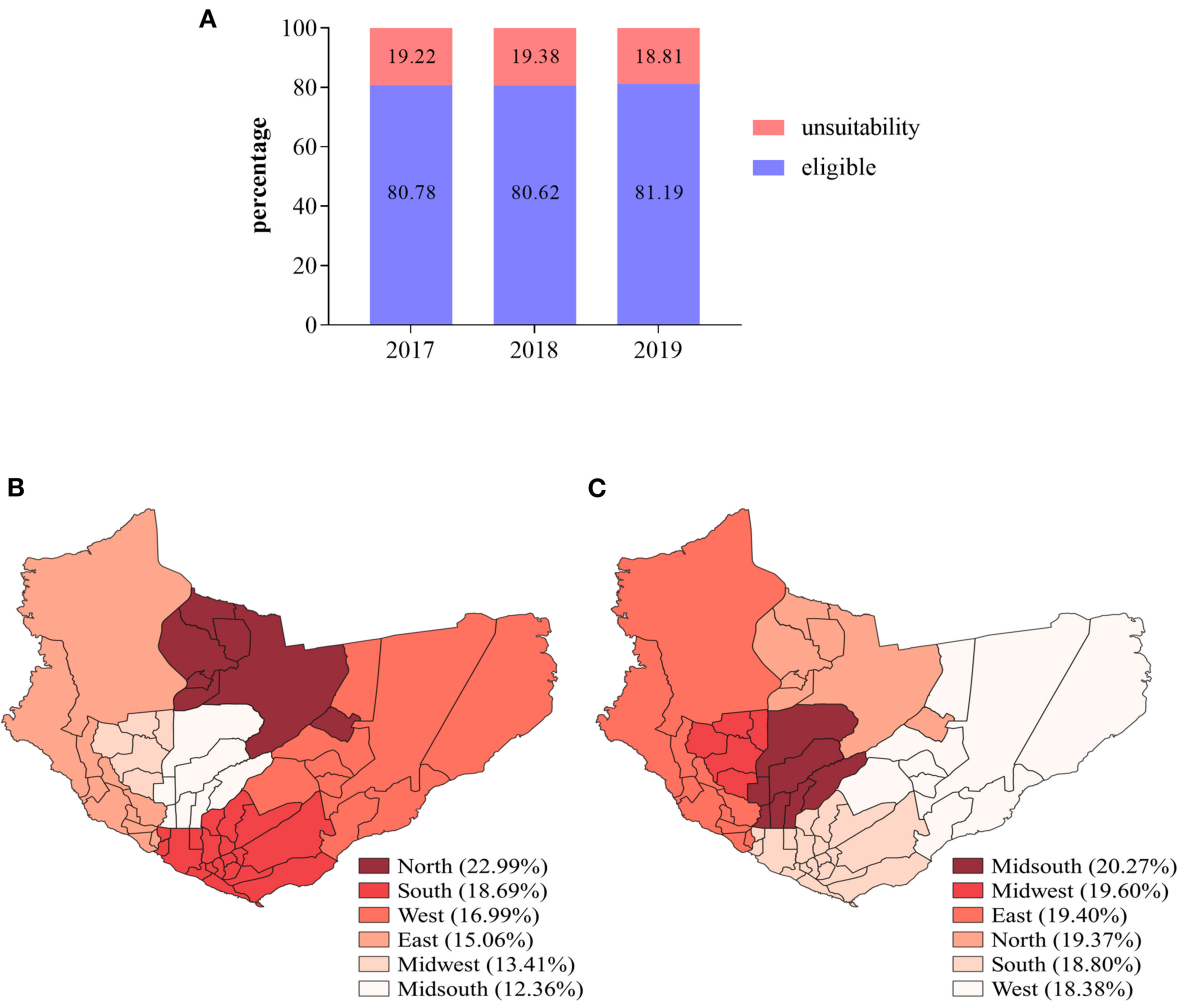
Total of suitable and unsuitable donors in the study population in the period 2017–2019. **(A)** Rates of suitable and unsuitable donors according to the year. **(B)** Site map showing the percentage of donors according to the area of the city of Manaus. **(C)** Site map showing unsuitability rates according to the area of the city of Manaus. Maps were built using the WGS1984 datum (worldclim software v.2.0).

The overall unsuitability rate for the triennium was 19.12% (*n* = 16,727). Total donors for 2017, 2018 and 2019 were 27,391 (31.32%), 28,855 (32.99%), and 31,217 (35.69%), respectively (Figure 1A). When unsuitability rates were analyzed according to sex, it was found that unsuitability was higher among women (28.86%; *p* < 0.00001) compared with men (14.70%). Of the total of 87,463 donors analyzed, about 99.03% were residents of the city of Manaus and 0.97% were residents of municipalities in the interior of the state. The north of Manaus concentrated the largest number of donors (22.99%), followed by the south (18.69%) and west (16.99%). The central-south had the lowest number of donors and the highest rate of unsuitability (Figures 1B, C). However, no significant difference was observed between unsuitability rates according to the areas of Manaus (Figure 1C).

Most of the sociodemographic variables analyzed were associated with unsuitability rates during first-time donations compared to return visits (Table 1). More than 80% of the study population consisted of individuals aged 20–49 years. The highest rates of unsuitability were observed among individuals belonging to the age groups of 16–19 years (21.59%) and 60–65 years (22.09%) (Table 1). When assessing the association between age and unsuitability, it was found that unsuitability increased in proportion to age, and the group of 60–65 years had the highest rates (68.63% of unsuitability in first-time donations and 20.43% in return visit donations). The results show that unsuitability was almost 6 times higher among individuals aged 60–65 years on the first visit (OR = 5.55; 95% CI 3.05–10.10; *p* < 0.0001) and 1.23 times on the return visit (OR = 1.23; 95% CI 1.05–1.44; *p* < 0.010), when compared to individuals aged 16–19 years (Table 1).

**Table 1:** Sociodemographic profile of unsuitable donors between the years 2017–2019.

					**First time**		**Return**	
**Sociodemographic profile**	**Total (%)**	**Total unsuitable (%)**	**Unsuitable first time (%)**	**Unsuitable return (%)**	**OR (CI 95%)**	***p*** **-value**	**OR (CI 95%)**	***p*** **-value**
**Sex**								
Male	60,139 (68.76)	8,840 (14.70)	2,130 (29.58)	6,710 (12.68)	Ref.		Ref.	
Female	27,324 (31.24)	7,887 (28.86)	2,641 (37.04)	5,246 (25.98)	1.40 (1.31–1.50)	<0.0001	2.42 (2.32–2.52)	<0.0001
**Age**								
16–19	5,363 (6.13)	1,158 (21.59)	594 (28.27)	564 (17.29)	Ref.		Ref.	
20–29	24,699 (28.24)	4,777 (19.34)	1,863 (29.20)	2,914 (15.91)	1.05 (0.94–1.17)	0.416	0.90 (0.82–1.00)	0.048
30–39	28,890 (33.03)	5,256 (18.19)	1,345 (36.27)	3,911 (15.53)	1.44 (1.29–1.62)	<0.0001	0.88 (0.80–0.97)	0.009
40–49	18,806 (21.50)	3,593 (19.11)	671 (43.54)	2,922 (16.92)	1.96 (1.70–2.25)	<0.0001	0.97 (0.88–1.08)	0.610
50–59	8,225 (9.40)	1,616 (19.65)	263 (47.64)	1,353 (17.63)	2.31 (1.91–2.80)	<0.0001	1.02 (0.92–1.14)	0.666
60–65	1,480 (1.69)	327 (22.09)	35 (68.63)	292 (20.43)	5.55 (3.05–10.10)	<0.0001	1.23 (1.05–1.44)	0.010
**Marital status**								
Single	43,775 (50.05)	8,904 (20.34)	3,004 (31.71)	5,900 (17.20)	Ref.		Ref.	
Common–law marriage	5,104 (5.84)	929 (18.20)	265 (33.76)	664 (15.37)	0.93 (0.79–1.09)	0.350	0.92 (0.84–1.01)	0.084
Married	35,129 (40.16)	6,146 (17.50)	1,353 (36.17)	4,793 (15.27)	0.92 (0.84–1.01)	0.094	0.91 (0.87–0.96)	<0.0001
Divorced	3,029 (3.46)	646 (21.33)	132 (44.44)	514 (18.81)	1.13 (0.88–1.44)	0.333	0.97 (0.87–1.07)	0.522
Widow(er)	426 (0.49)	102 (23.94)	17 (47.22)	85 (21.79)	0.96 (0.49–1.89)	0.907	0.92 (0.72–1.19)	0.537
**Education**								
Elementary school	6,010 (6.87)	1,184 (19.70)	259 (37.16)	925 (17.41)	Ref.		Ref.	
High school	44,257 (50.60)	8,217 (18.57)	2,295 (32.72)	5,922 (15.90)	0.94 (0.79–1.10)	0.437	0.89 (0.82–0.96)	0.003
Incomplete higher education	14,737 (16.85)	3,030 (20.56)	1,139 (32.87)	1,891 (16.78)	1.03 (0.87–1.23)	0.702	0.87 (0.79–0.95)	0.003
Complete higher education	20,707 (23.68)	3,920 (18.93)	979 (33.95)	2,941 (16.50)	0.84 (0.71–1.00)	0.056	0.81 (0.74–0.88)	<0.0001
Post–graduate	1,752 (2.00)	376 (21.46)	99 (36.40)	277 (18.72)	0.86 (0.64–1.15)	0.315	0.84 (0.72–0.98)	0.024
**Occupation**								
Administration/Accountancy	0.5010 (5.73)	1,021 (20.38)	275 (35.48)	746 (17.62)	Ref.		Ref.	
Retired	465 (0.53)	85 (18.28)	13 (61.90)	72 (16.22)	1.39 (0.54–3.58)	0.490	0.85 (0.65–1.12)	0.262
Self–employed	9,724 (11.12)	2,268 (23.32)	711 (34.62)	1,557 (20.30)	0.90 (0.76–1.08)	0.265	1.12 (1.01–1.23)	0.030
Merchant	4.267 (4.88)	913 (21.40)	286 (37.93)	627 (17.85)	1.08 (0.87–1.34)	0.493	1.13 (1.00–1.28)	0.048
Education	13,710 (15.68)	3,094 (22.57)	1,219 (32.14)	1,875 (18.91)	1.02 (0.86–1.21)	0.814	1.06 (0.96–1.17)	0.265
Industry	5,319 (6.08)	888 (16.69)	210 (32.21)	678 (14.53)	0.94 (0.75–1.17)	0.557	0.99 (0.89–1.12)	0.930
Information technology	1,038 (1.19)	154 (14.84)	34 (23.29)	120 (13.45)	0.64 (0.43–0.97)	0.037	1.00 (0.81–1.24)	0.991
Law	1,048 (1.20)	220 (20.99)	51 (31.88)	169 (19.03)	0.85 (0.59–1.22)	0.373	1.14 (0.95–1.38)	0.170
Mechanical/Civil engineering	3,765 (4.30)	586 (15.56)	131 (32.67)	455 (13.53)	0.96 (0.74–1.24)	0.748	0.99 (0.87–1.13)	0.899
Health	4,354 (4.98)	921 (21.15)	234 (38.30)	687 (18.35)	1.01 (0.81–1.26)	0.925	0.87 (0.77–0.98)	0.020
Security	2,685 (3.07)	394 (14.67)	48 (30.38)	346 (13.69)	0.93 (0.75–1.14)	0.480	0.95 (0.85–1.05)	0.296
Civil service	12,187 (13.93)	1,872 (15.36)	377 (31.18)	1,495 (13.62)	0.91 (0.70–1.17)	0.459	0.91 (0.81–1.01)	0.083
Transport	4,171 (4.77)	654 (15.68)	118 (37.46)	536 (13.90)	1.14 (0.87–1.51)	0.339	1.05 (0.93–1.19)	0.445
Other	19,720 (22.55)	3,657 (18.54)	1,064 (32.40)	2,593 (15.78)	0.94 (0.80–1.11)	0.480	1.04 (0.95–1.14)	0.405
**Motivation**								
Spontaneous	33,493 (38.29)	6,358 (18.98)	1,437 (36.79)	4,921 (16.63)	Ref.		Ref.	
Reposition	49,772 (56.91)	9,473 (19.03)	3,041 (31.60)	6,432 (16.02)	0.77 (0.71–0.83)	<0.0001	0.97 (0.93–1.01)	0.168
Campaign	2,463 (2.82)	538 (21.84)	173 (36.34)	365 (18.37)	0.99 (0.81–1.21)	0.933	1.06 (0.94–1.19)	0.375
Educational institution	462 (18.76)	94 (20.35)	43 (24.43)	51 (17.83)	Ref.		Ref.	
Religious institution	1,465 (59.48)	352 (24.03)	103 (48.82)	249 (19.86)	2.75 (1.76–4.31)	<0.0001	1.23 (0.87–1.73)	0.237
Other campaigns	536 (21.76)	92 (17.16)	27 (30.34)	65 (14.54)	1.33 (0.74–2.40)	0.338	0.82 (0.54–1.23)	0.337
Other motivation	1,735 (1.98)	358 (20.63)	120 (36.59)	238 (16.92)	1.05 (0.83–1.34)	0.666	0.98 (0.85–1.13)	0.752

OR, Odds ratio; CI, Confidence interval; OR and CI were calculated from the logistical regression analysis. Marital status, Education, Occupation and Motivation were adjusted by Age and Gender.

When observing the marital status of the donors, it was found that singles constitute the majority (50.05%), with a higher unsuitability rate (20.34%) than those in a common-law marriage (18.20%) or married (17.50%). Widows/widowers, in turn, corresponded to a total of 0.49% of donors, yet they presented the highest rates of unsuitability, both on the first visit (47.22%) and on the return visit (21.79%). Regarding marital status, only married individuals were less likely to be unsuitable, during return donation (OR = 0.91; 95% CI 0.87–0.96; *p* < 0.0001).

In the evaluation of unsuitability rates according to the level of education, although the largest share of donors came from individuals with secondary education, the unsuitability rates were lower for this group both on the first visit (32.72%) and on the return visit (15.90%). No statistically significant association was observed between unsuitability and the level of education during the first donation. However, during the return visit, the unsuitability was directly correlated with the educational level. The unsuitability tended to be lower in individuals with high school (OR = 0.89; 95% CI 0.82–0.96; *p* = 0.003), incomplete higher education (OR = 0.87; 95% CI 0.79–0.95; *p* = 0.003) and complete higher education (OR = 0.82; 95% CI 0.74–0.88; *p* < 0.0001).

Regarding professional occupations, it was observed that the highest rates occurred among individuals from the areas of education (15.68%), civil service (13.93%) and the self-employed (11.12%). The lowest percentage of unsuitability were observed among individuals from the areas of administration/accountancy, merchants, law and health. Although the number of retired participants was the lowest among the groups of the study population (n = 465), this group presented the highest rate of unsuitability (61.90%). The occupations self-employed (OR = 1.12; 95% CI 1.01–1.23; *p* = 0.030) and merchant (OR = 1.13; 95% CI 1.00–1.28; *p* = 0.048) were more related to unsuitability during return donation, while health workers were less likely to be unsuitable (OR = 0.87; 95% CI 0.77–0.98; *p* = 0.020). In addition, even though few individuals work in the information technology field (1.19%), this occupation was associated with the lowest probability of unsuitability (OR = 0.64; 95% CI 0.43–0.97; *p* = 0.037).

The largest share of donations, according to motivation, happened through replacement donations (49,772; 56.91%), followed by spontaneous donations (33,493; 38.29%) and campaigns (2,463; 2.82%), with a higher rate of unsuitability in the first donation (Table 1). Blood donation campaigns are targeted at institutions and organized groups. Only the donations from campaigns carried out by religious institutions showed a significant association with unsuitability (OR = 2.75; 95% CI 1.76–4.31; *p* < 0.0001) during the first donation, and these are almost three times more likely to result in unsuitable donations.

### 3.2. Main causes of unsuitability observed during donor screening

**Figure 2:**
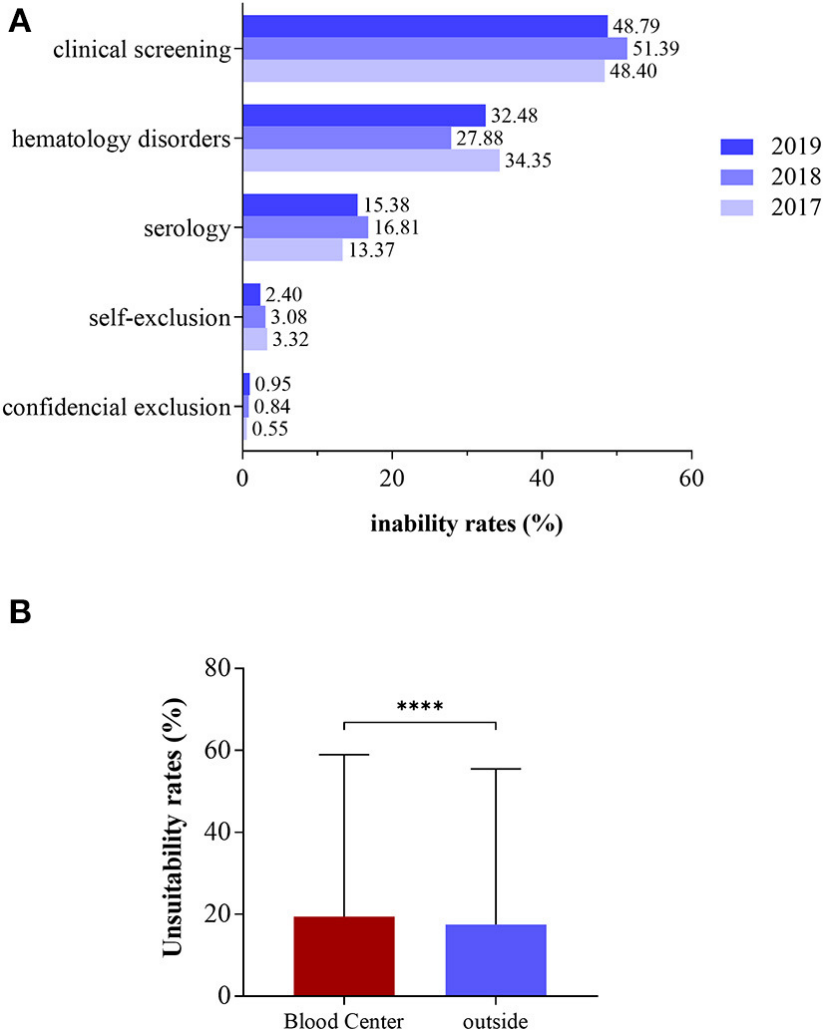
Causes of unsuitability according to year **(A)** and place of donation **(B)**. ****p < 0.00001 (Student's *t*-test).

**Figure 3:**
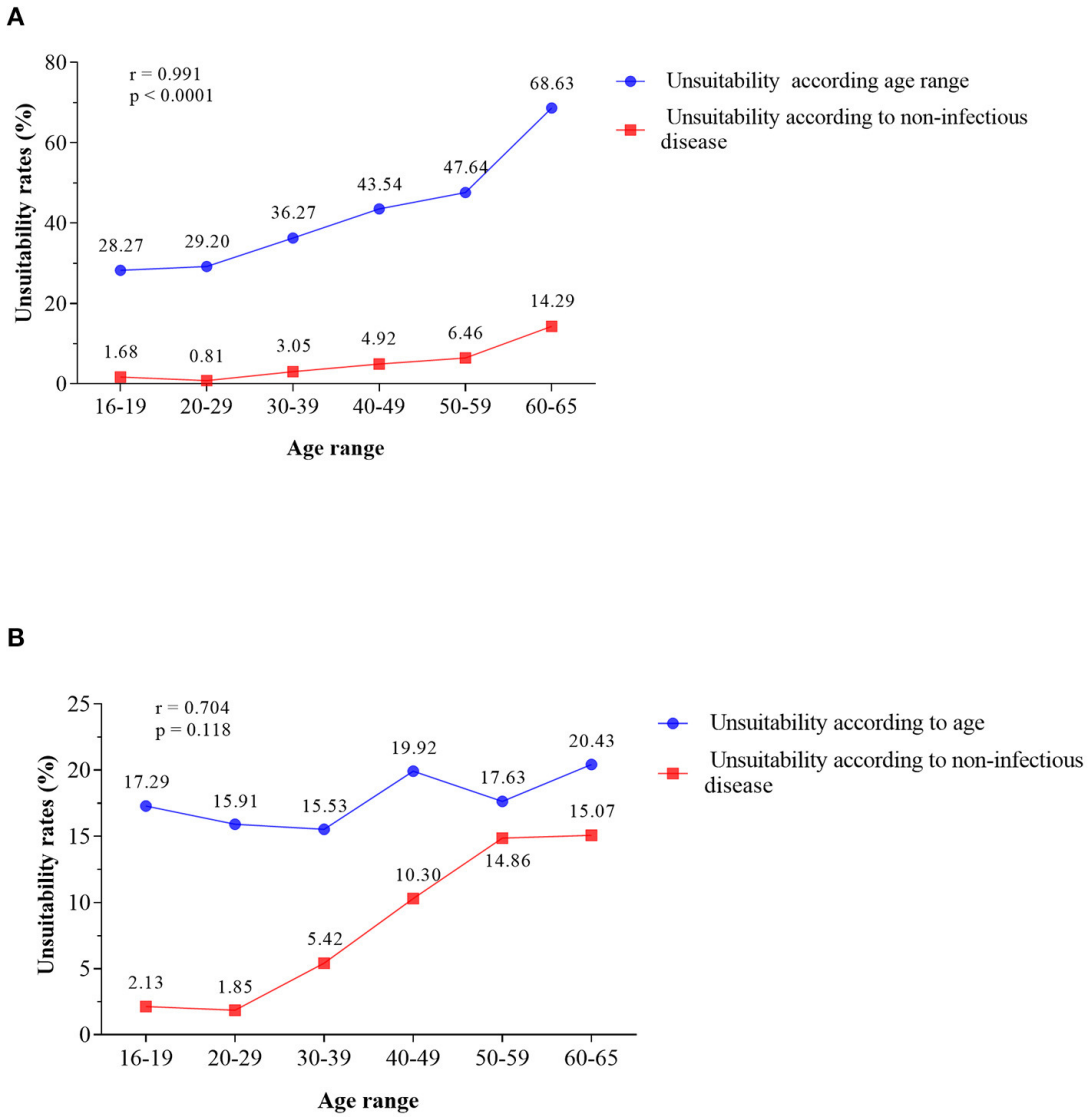
Positive correlation between the increase in donor age and the percentage of unsuitability due to chronic non-infectious diseases. **(A)** First donation and return visit. **(B)** Pearson's correlation.

The clinical screening was responsible for an unsuitability rate that ranged from 48.40 to 51.39% during the triennium, while unsuitability rates due to hematological disorders ranged from 27.88 to 34.35% (Figure 2A). Self-exclusion and confidential exclusion resulted in lower unsuitability rates. When the unsuitability rates were evaluated according to the collection site, the HEMOAM blood bank showed a higher rate (19.40%) when compared to the external collection stations (17.50%), with a statistically significant difference (*p* < 0.00001) (Figure 2B). In the correlation analysis between donor unsuitability and chronic non-infectious diseases in relation to age, it was observed that there is a strong positive correlation (r >0.7) that is statistically significant only for the first donation (Figure 3A, r = 0.991; *p* < 0.0001). On the other hand, there was no significant statistical correlation for the return visit (Figure 3B, r = 0.704; *p* = 0.118).

Inadequate nutritional status (15.17%), non infectious chronic diseases (11.40%), sexual habits (9.5%), risk exposure (8.83%), surgical and invasive procedures (7.62%) and medication use (7.57%) were the main causes of unsuitability during donor screening. These variables were responsible for 60.09% of unsuitability (Table 2). Regarding inadequate nutritional status, the donor who had not had lunch was the main reason for unsuitability both on the first visit (51.23%) and on the return visit (50.44%), while sex with multiple partners was the main cause of unsuitability (32.15%) among those who were unsuitable due to risk behavior (Table 3). Living in or having been in an endemic area for malaria accounted for more than 99% of the unsuitability among those who were unsuitable due to risk exposure. This situation is understandable considering that malaria is an endemic disease in the state of Amazonas, including in some urban areas of Manaus.

**Table 2:** Reasons for being unsuitable during clinical screening in the 2017–2019 triennium.

**Reason for being clinically unsuitable**	**Total (%)**	**First time (%)**	**Return (%)**
Nutritional status	1,257 (15.17)	454 (20.56)	803 (13.21)
Non-infectious chronic diseases	945 (11.40)	121 (5.48)	824 (13.56)
Sexual habits	787 (9.50)	264 (11.96)	523 (8.60)
Exposure to risk	732 (8.83)	169 (7.65)	563 (9.26)
Surgical or invasive procedures	631 (7.62)	182 (8.24)	449 (7.39)
Use of medication	627 (7.57)	156 (7.07)	471 (7.75)
Withdrew	611 (7.37)	96 (4.35)	515 (8.47)
Infectious diseases	450 (5.43)	152 (6.88)	298 (4.90)
Vaccine	434 (5.24)	69 (3.13)	365 (6.01)
Dermatological diseases	319 (3.85)	62 (2.81)	257 (4.23)
Inflammatory diseases	277 (3.34)	97 (4.39)	180 (2.96)
Insomnia	268 (3.23)	115 (5.21)	153 (2.52)
Travel	191 (2.31)	50 (2.26)	141 (2.32)
Underweight	187 (2.26)	72 (3.26)	115 (1.89)
Other	570 (6.88)	149 (6.75)	421 (6.93)

**Table 3:** Reasons for being unsuitable during clinical screening stratified according to non-disease-related variables in the 2017–2019 triennium.

**Behavior\condition**	**Total unsuitable (%)**	**Subgroup**	***N* (%)**	**First time (%)**	**Return (%)**
Nutritional status		Fasting	428 (34.05)	150 (33.04)	278 (34.62)
	15.17	Did not eat lunch	644 (51.23)	239 (52.64)	405 (50.44)
		Did not eat in the last 2 h	185 (14.72)	65 (14.32)	120 (14.94)
Sexual habits		Multiple partners with condom	476 (60.48)	145 (54.92)	331 (63.29)
	9.50	Multiple partners without condom	253 (32.15)	86 (32.58)	167 (31.93)
		Same–sex partners	51 (6.48)	29 (10.98)	22 (4.21)
		Sex with someone suspected of HIV	7 (0.89)	4 (1.52)	3 (0.57)
Exposure to risk		Contact with hepatitis carrier	4 (0.55)	1 (0.59)	3 (0.53)
	8.83	Visited malaria zone	728 (99.45)	168 (99.41)	560 (99.47)
Use of medication		Antiallergic	32 (5.10)	9 (5.77)	23 (4.88)
		Antibacterial	80 (12.76)	17 (10.90)	63 (13.38)
		Anticoagulants	4 (0.64)	1 (0.64)	3 (0.64)
		Antifungal	35 (5.58)	4 (2.56)	31 (6.58)
		Anthelminthics	28 (4.47)	5 (3.21)	23 (4.88)
		Antihypertensive	47 (7.50)	7 (4.49)	40 (8.49)
	7.57	Antipsychotic	16 (2.55)	6 (3.85)	10 (2.12)
		Corticosteroid	15 (2.39)	5 (3.21)	10 (2.12)
		Finasteride	11 (1.75)	1 (0.64)	10 (2.12)
		Isotretinoin	8 (1.28)	1 (0.64)	7 (1.49)
		Hormone replacement	10 (1.59)	1 (0.64)	9 (1.91)
		Not specified	341 (54.39)	99 (63.45)	242 (51.39)
Surgical or invasive procedures		Surgery	131 (21.00)	33 (18.00)	98 (22.00)
		Acupuncture	12 (2.00)	2 (1.00)	10 (2.00)
	7.62	Piercing	45 (7.00)	22 (12.00)	23 (5.00)
		Pierced ear	25 (4.00)	9 (5.00)	16 (4.00)
		Tattoo	204 (32.00)	71 (39.00)	133 (30.00)
		Not specified	214 (34.00)	45 (25.00)	169 (37.00)

In relation to diseases, non-infectious chronic diseases, infectious diseases, inflammatory diseases and dermatological diseases, contributed to 24.02% of the unsuitability during the clinical screening of the donor, and may be responsible for permanent unsuitability (Table 2). Among infectious diseases, viral infection accounted for 93.3% of unsuitability (Supplementary Table S1). In the category of inflammatory diseases, sore throat, sinusitis, fever and tonsillitis or otitis, combined, accounted for 85.19% of unsuitability.

Dermatological conditions, lesions (boils, infected pimples, rashes, etc.) represented 60% of unsuitability (Supplementary Table S1). Hypertension was the reason for 85.19% of unsuitability (Supplementary Table S1). Of the 34 individuals who were unsuitable due to diabetes, 29 were unsuitable only on the return visit, which indicates that this chronic disease developed or was diagnosed after the first donation.

## 4. Discussion

Characterizing and understanding the factors related to the profile of unsuitable donors during blood donation remains the best alternative to follow the changes in the pattern of this prosocial behavior. In this sense, this study describes the clinical and sociodemographic profile of unsuitability for blood donation in the state of Amazonas during the period 2017–2019.

The rate of general unsuitability observed for the triennium studied was 19.12%, which is a value that is similar to the general rates observed in Brazil in 2018 (19.46%) and 2019 (18.87%) (13). Most of the donations came from the city of Manaus, and the central-south area was responsible for the highest rate of unsuitability and the lowest number of donors as well, which is interesting since it concentrates the highest per capita income in the state (14). In Tanzania, high-income cities have higher donation rates compared to low-income ones (15). Also, studies on the profile of blood donors in the Brazilian population show that the act of donating blood tends to be higher among young men with higher purchasing power and high educational level (16, 17). These studies demonstrate that blood donation suitability and donation volume tend to be concentrated in regions with higher per capita income. However, our findings demonstrated that most of the blood donors from Amazonas state were single male individuals between 30–39 years of age, with complete high school education, living in the most populous areas of Manaus.

In contrast, the highest rates of unsuitability were observed among women of all ages. People aged 60–65 years, self-employed and merchant are more likely to be unsuitable. The unsuitability was more frequent in the first donation than in the return visit. As in the Amazonas state, in Iran, Nepal, Nigeria and India, the lowest donation rates and the highest rates of unsuitability are also observed among women (18–21). It is important to emphasize that there are factors that contribute to unsuccessful donations related to the female, such as menstruation, pregnancy, and breastfeeding, among other factors (22, 23). However, the available data were not sufficient to determine the causes that resulted in the high rates of unsuitability among women observed in the study population.

As for the place of donation, there was a greater number of donations and unsuitability in the blood center than in external campaigns. However, when the donation was considered based on the type of motivation, campaign performance was associated with higher rates of unsuitability (21.84%) compared to voluntary and replacement donations. Campaigns are of increasing importance in the retention of volunteers (24). Their main objective is to raise awareness among the population, strengthen the continuity of donations and encourage the spirit of solidarity (25). The data presented in this study indicate the need and importance of the implementation of educational actions of dissemination, information and guidance directed mainly toward awareness and preparation for blood donation, to reduce the number of unsuitable candidates during the clinical screening of the donor that is carried out during the campaigns.

Our results showed that the average rate of unsuitability during donor clinical screening was 49.52% in the triennium studied. In Europe, the United States and Canada, unsuitability rates average approximately 10%, with a significant variation of 1.4 to 25% (26). In Brazil, according to the latest bulletin of the National Agency for Health Surveillance (ANVISA) regarding hemotherapy production (HEMOPROD), the combined causes of unsuitability during clinical screening correspond to 35.98%, and most correspond to “unknown causes” (13). In the present study, inadequate nutritional status was the main cause of unsuitability during clinical screening. This fact deserves attention because the nutritional condition, as a cause of unsuitability, does not appear in the HEMOPROD bulletin. This may be a reflection of the socio-economic status of the study population or the low level of knowledge about the appropriate preparation for blood donation. However, this factor needs to be analyzed in more detail to identify the reasons underlying the low nutritional status of the study population observed at the time of donation.

The hematological screening was the second leading cause of unsuitability in this study. However, in Brazil, hematological screening is the first cause of unsuitability, according to HEMOPROD (13). Low hemoglobin is a factor of unsuitability with a high incidence in several populations in different parts of the world, and is more common in women than men (27). Low hemoglobin levels are related to increasing age, high temperatures, low body weight, low income, poor diet, and short intervals between donations, and there is a higher frequency among Hispanic or Afro-descendant donors (28). Most donors in the state of Amazonas are “mestiço” (individuals with various ethnic backgrounds) living in populated areas, which may have a direct relationship with the rates of unsuitability resulting from hematological screening.

Among the types of unsuitability due to infectious diseases, viral infection was the main cause. A similar situation has also been described in other regions of Brazil and many countries in Europe, which have demonstrated that viral infections are usually the main causes of unsuitability for infectious diseases, especially in first-time donations (29, 30). The state of Amazonas is an endemic region for several viral and parasitic diseases, such as arboviruses and malaria, which can directly influence these rates (31, 32). Almost 100% of the unsuitability due to risk exposure were due to the individual having been in an endemic area for malaria.

Concerning chronic diseases, the main cause of unsuitability was hypertension. There is a relationship between the cumulative incidence, age and the first occurrence of chronic disease, and its onset or emergence is usually higher in women than in men (33). In Spain, it was observed that the number of able-bodied donor women gradually decreases between the ages of 30 and 50, and men follow this decline from the age of 50 (34). In India, hypertension was the main chronic disease responsible for unsuitability in the donor age group between 56 and 65 years (18). Our findings demonstrated a statistically significant positive correlation between increasing age and the frequency of chronic diseases in the study population, which could explain the increasing unsuitability with the observed advancing age.

This study presented some limitations, such as the absence of more detailed data, insufficient data for 2020 (which would allow assessing the impact of the pandemic on donation/unsuitability rates) and the transversality of the analyses. However, the scenario presented by this study is directly related to the geographical and sociodemographic particularities of the Amazon region. In addition, the predominance of replacement donations that was observed may be directly associated with factors related to unsuitability during clinical screening, such as inadequate nutritional status.

In summary, this study described the main factors associated with donor unsuitability in the state of Amazonas, many of which are peculiar to the Amazon region. The characterization of the profile of suitable and unsuitable candidates made through this study will allow a better understanding of the panorama of blood donation in the Brazilian Amazon. Also, our findings will serve as a basis for the implementation of strategic actions and campaigns that reduce the rates of unsuitability and encourage the return visit of candidates with temporary postponement for a new donation and subsequent retention.

## Data availability statement

The raw data supporting the conclusions of this article will be made available by the authors, without undue reservation.

## Ethics statement

The studies involving human participants were reviewed and approved by the Research Ethics Committee (REC) of the Amazonas State Hematology and Hemotherapy Foundation (HEMOAM) (CAEE 39449920.3.0000.0009). The patients/participants provided their written informed consent to participate in this study.

## Author contributions

Conceptualization: GP and GR. Methodology and investigation: GR and AB. Formal analysis: GP, GR, and AB. Validation, resources, data curation, and writing-review and editing: GP and AB. Writing-original draft preparation: GR. Supervision, project administration, and funding acquisition: GP. All authors have read and agreed to the final version of the manuscript.
