# Supplementary material for: Trends in unsuitability for blood donation in the Brazilian Amazon
Source: Front Public Health. 2022 Dec 22;10:1056332. doi: 10.3389/fpubh.2022.1056332 (PMC9814009; doi:10.3389/fpubh.2022.1056332)
Supplement: Supplementary file 1 [file Table_1.DOCX]

**Supplementary Table 1.** Reasons for being unsuitable during clinical screening stratified according to the presence of infectious and non-infectious diseases in the 2017-2019 triennium.

| **Group of diseases or condition** | **Disease, condition or cause** | **Total (%)** | **First time (%)** | **Return (%)** |
| --- | --- | --- | --- | --- |
| Infectious | Bacterial | 20 (4.44) | 4 (2.63) | 16 (5.37) |
|  | Protozoa | 9 (2.00) | - | 9 (3.02) |
|  | Viral | 420 (93.33) | 148 (97.37) | 272 (91.28) |
|  | Other | 1 (0.23) | - | 1 (0.34) |
| Inflammatory | Asthma or bronchitis | 7 (2.53) | 4 (4.12) | 3 (1.67) |
|  | Conjunctivitis | 9 (3.25) | - | 9 (5.00) |
|  | Migraine/Headache | 25 (9.03) | 8 (8.25) | 17 (9.44) |
|  | Fever | 83 (29.96) | 33 (34.02) | 50 (27.78) |
|  | Sore throat | 109 (39.35) | 38 (39.18) | 71 (39.44) |
|  | Sinusitis, tonsillitis or otitis | 44 (15.88) | 14 (14.43) | 30 (16.67) |
| Dermatological | Allergy | 113 (35.42) | 24 (38.71) | 89 (34.63) |
|  | Lesions | 194 (60.82) | 34 (54.84) | 160 (62.26) |
|  | Psoriasis | 12 (3.76) | 4 (6.45) | 8 (3.11) |
| Chronic non-infectious | Cancer | 6 (0.63) | 2 (1.65) | 4 (0.49) |
|  | Diabetes | 34 (3.60) | 5 (4.13) | 29 (3.52) |
|  | Neurological/mental illness | 6 (0.63) | 1 (0.83) | 5 (0.61) |
|  | Rheumatic diseases | 6 (0.63) | 1 (0.83) | 5 (0.61) |
|  | Hypercholesterolemia | 57 (6.03) | 15 (12.40) | 42 (5.10) |
|  | Hypertension | 805 (85.19) | 87 (71.90) | 718 (87.14) |
|  | Hypotension | 31 (3.29) | 10 (8.26) | 21 (2.55) |
